# Medical Cannabis as Adjunctive Therapy for Head and Neck Cancer Patients

**DOI:** 10.7759/cureus.18396

**Published:** 2021-09-30

**Authors:** Mathew P Caputo, Carmen S Rodriguez, Tapan A Padhya, Matthew J Mifsud

**Affiliations:** 1 Otolaryngology - Head and Neck Surgery, University of South Florida Morsani College of Medicine, Tampa, USA; 2 College of Nursing, University of South Florida, Tampa, USA

**Keywords:** chemotherapy induced nausea and vomiting (cinv), cancer pain, head & neck cancer, cannabinoid, cannabis

## Abstract

The goal of this systematic review was to define a consensus within the current literature regarding the impact/effect of cannabis or cannabinoids on the treatment of patients with head and neck cancer. We conducted a review of PubMed, Embase, and Web of Science databases, using a comprehensive search strategy, focusing on articles relating to head & neck cancer and cannabis/cannabinoids without a time limit for publication.

Two, independent reviewers screened articles based on title/abstract and included the ones selected by both. We then conducted a full-text review and excluded all articles which did not meet inclusion criteria. A single reviewer then assessed studies for methodological quality and extracted relevant data using a premade data collection tool. We identified five studies that met inclusion criteria. Studies were of varying quality and the majority investigated recreational cannabis use with only one study reporting dosing across participants. Lack of standardized cannabis exposure presents a wide array of potential confounding variables across the remaining studies. Meta-analysis was not attempted due to variability in reported outcomes.

It is impossible to draw any conclusions regarding the benefit or adverse effects of current medical cannabis products in this patient population. The literature regarding the effect of cannabis/cannabinoids on head & neck cancer patients is limited. However, the current lack of evidence does not definitively disprove the efficacy of cannabis. High-quality studies are necessary for physicians to provide advice to patients who are either using or interested in cannabis as an adjunctive treatment.

## Introduction and background

On November 5, 1996, the state of California enacted the Compassionate Use Act of 1996 making it the first state to allow the medical use of cannabis since it was federally prohibited in 1937 [1,2]. At a state level, cannabis policy has continued to relax despite continued federal classification as a Schedule I controlled substance [2-4].

The term Cannabis typically refers to the *Cannabis sativa* plant, while cannabinoids refer to a diverse group of active compounds produced naturally or artificially [5,6]. Medicinal cannabis can thus be found in many forms, with varying levels of the two main therapeutic cannabinoids, tetrahydrocannabinol (THC) and cannabidiol (CBD) [7]. A variety of medicinal cannabis products are outlined in Table 1. There is evidence to suggest an effect of cannabinoids for the management of various symptoms associated with cancer treatment; particularly pain, nausea/vomiting, and cachexia [8,9]. However, the legal status of cannabis has limited clinical research. Thus, literature analyses querying the utility of medicinal cannabis generally include low-quality data [8,9].

**Table 1:** Cannabis Products FDA=Food and Drug Administration, THC= Therapeutic Cannabinoids Tetrahydrocannabinol, CBD= Cannabidiol, AIDS= Acquired Immunodeficiency Syndrome References [10-13]

Class	Name	THC: CBD	Routes of Administration	Approval
Synthetic:	dronabinol (Marinol, Syndros)	Synthetic THC	Oral	FDA approved as second-line therapy for chemotherapy-induced nausea and vomiting and AIDS-related anorexia and wasting.
	nabilone (Cesamet)	Synthetic THC analog	Oral	FDA approved as second-line therapy for chemotherapy-induced nausea and vomiting and AIDS-related anorexia and wasting.
Phytocannabinoids:	Herbal cannabis	Variable	Inhalation (smoked/vaporized) Ingested	Not FDA approved.
	Cannabis extraction products	Variable	Inhalation (smoked/vaporized) Ingested (oil/pill) Topical	Not FDA approved.
	nabiximols (Sativex®)	1:1	Oromucosal spray	Not FDA approved. Approved in Canada, Israel, and several European countries for the treatment of multiple sclerosis-related muscle spasticity.

Recent observational studies have demonstrated an increased incidence of cannabis use among current cancer patients (18%-30%) [14-16]. Despite limited clinical data, increased legality and cultural acceptance are driving this growing utilization amongst cancer patients, presumably including those under treatment for head & neck malignancies [17]. There has not yet been a systematic analysis of the literature investigating the efficacy of medicinal cannabis as a supportive therapy amongst head & neck cancer patients. The goal of this review is to define a consensus among the current literature regarding the safety and efficacy of medicinal cannabis products in this patient population. 

## Review

Methods

Preferred Reporting Items for Systematic Reviews and Meta-Analyses (PRISMA) 2020 guideline for reporting systematic reviews was followed for this systematic review article (Appendix A). This review was not previously registered, but the protocol is provided in the supplemental materials (Appendix B). We conducted a search of PubMed, Embase, and Web of Science using a comprehensive search strategy (Appendix C). The search strategy focused on all articles relating to both head & neck cancer, cannabis, or any cannabinoid-derived medication without any time limit for publication. Two independent reviewers screened manuscripts based on title/abstract to identify those that detailed the potential therapeutic application of medicinal cannabis for cancer/treatment specific symptoms. Only English language, peer-reviewed literature was considered for this search.

Only manuscripts selected by both reviewers were considered for the next stage of the review. Subsequently, two reviewers conducted a full-text analysis. At this stage, we excluded all manuscripts that did not detail the therapeutic application of medicinal cannabis products in head & neck cancer patients. Disagreements between reviewers were settled through discussion. Using a modified National Institutes of Health Study Quality Assessment Tool, a single reviewer assessed the methodological quality of included studies and ranked them on a scale of zero to eleven. A higher rating indicates a higher quality study [18]. After selection and quality assessment, a single reviewer extracted any data related to the efficacy of cannabis in treating the symptoms of head & neck cancer and its treatments, along with data pertaining to the tolerability of cannabis products. Other data collected included study design, population size, patient demographics (i.e., cancer type, treatment status, treatment type), type of cannabis/cannabinoid product, and dosage of cannabis/cannabinoids. All data were collected using a premade data collection template. 

Additionally, we queried OpenGrey, WONDER, and ClinicalTrials.gov for any additional “gray literature” using a similar search strategy.

Results

**Figure 1:**
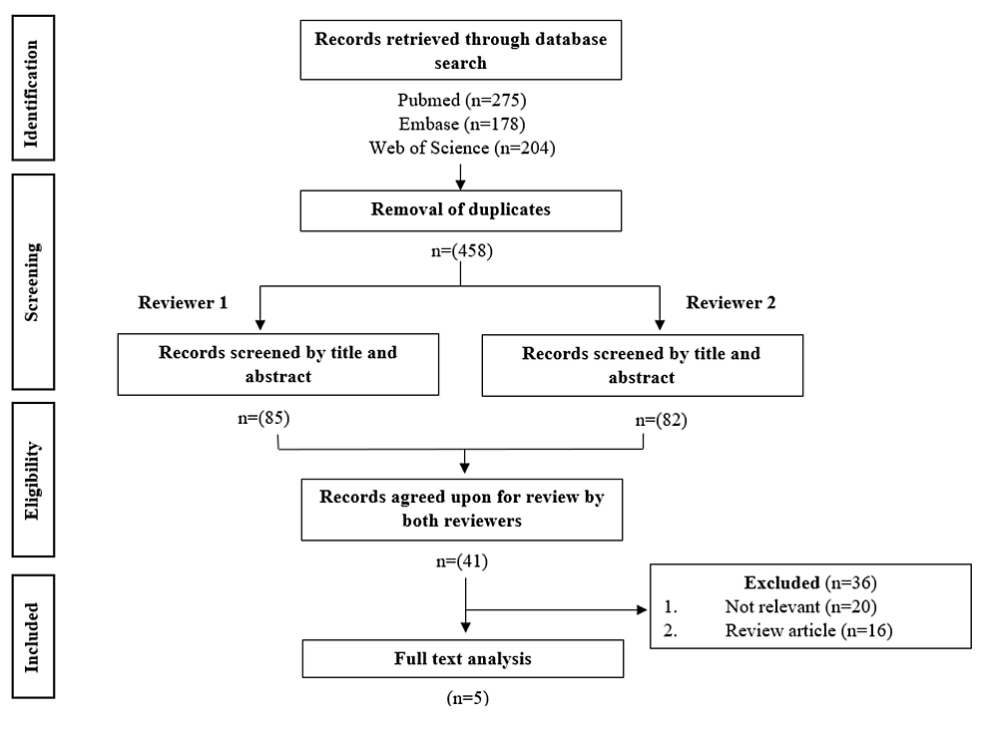
PRISMA flow diagram detailing inclusion process PRISMA - Preferred Reporting Items for Systematic Reviews and Meta-Analyses (https://doi.org/10.1136/bmj.n71)

Our search yielded 275 studies from PubMed, 178 from Embase, and 204 from Web of Science. After removal of duplicate articles and screening by title/abstract, we selected 41 articles for full-text review. Following full-text analysis, we identified only five studies that provided relevant information and met inclusion criteria. This screening process is displayed in more detail in Figure 1. Unfortunately, our query of OpenGrey and WONDER did not yield any relevant pieces of “gray literature.” However, we were able to find a single relevant, ongoing study through ClinicalTrials.gov.

The quality of studies identified varied, ranging from four to eleven on the Study Quality Assessment Tool (Table 2). As far as study design, our search found two prospective cohort studies, one retrospective cohort study, one cross-sectional survey, and one randomized controlled trial (RCT). One study identified was abstract-only as opposed to a full-text article [19]. Additionally, it should be noted that two of these studies had an overlapping population but were included as they both analyzed separate outcomes within the same patient population [20,21].

**Table 2:** Study Quality Assessment References [19-23]

Source	Research question clearly stated?	Population clearly defined?	Subjects recruited from same/similar populations?	Inclusion and exclusion criteria clearly defined?	Sample size justification, power description, or variance estimates provided?	Exposures of interest measured prior to outcomes?	Exposure measures clearly defined?	Outcome measures clearly defined?	Confounding variables adjusted for statistically?	Statistical methods well-described?	Results well described?	Total
Ghanem et al. 2020 [19]	Yes	Yes	Yes	No	No	No	No	No	No	No	Yes	4/11
Zhang et al. 2019 [20]	Yes	Yes	Yes	Yes	Yes	Yes	No	Yes	Yes	Yes	Yes	10/11
Zhang et al. 2018 [21]	Yes	Yes	Yes	Yes	Yes	Yes	No	Yes	Yes	Yes	Yes	10/11
Elliot et al. 2016 [22]	Yes	Yes	Yes	Yes	No	Yes	No	No	No	No	No	5/11
Cote et al. 2015 [23]	Yes	Yes	Yes	Yes	Yes	Yes	Yes	Yes	Yes	Yes	Yes	11/11

A summary of study characteristics, conclusions, and notable limitations is depicted in Table 3. A meta-analysis was not attempted due to the low number of appropriate studies identified and the variance in data reported in each. 

**Table 3:** Study Results OPSCC=Oropharyngeal Squamous Cell Carcinoma, HNSCC=Head and Neck Squamous Cell Carcinoma, HNC=Head and Neck Cancer, OMMP=Oregon Medical Marijuana Program, PO=orally, BID=twice daily, QID=four times daily References  [19-23]

Source	Population	Population Size	Cannabis/Cannabinoid Exposure	Dose	Outcomes
Ghanem et al. 2020 [19]	OPSCC patients undergoing treatment	74	Recreational cannabis use	N/A	Cannabis use was associated with significantly worse overall survival but not with significantly different disease free state, locoregional recurrence, distant metastasis pain score or weight loss during radiotherapy.
Zhang et al. 2019 [20]	P16 positive OPSCC patients undergoing treatment with curative intent	94	Recreational, loose-leaf cannabis use	N/A	No significant difference in survival, disease recurrence, distant metastasis between marijuana and non-marijuana users within this population. No evidence that marijuana leads to adverse events observed in this population.
Zhang et al. 2018 [21]	HNSCC patients undergoing treatment with curative intent	148	Recreational, loose-leaf cannabis use	N/A	Recreational use of loose-leaf marijuana was associated with statistically significant improvement in patient reported pain, anxiety, depression and general well-being. No difference in mobility, self-care or usual activity scores.
Elliot et al. 2016 [22]	HNC patients enrolled in OMMP	15	Medical cannabis use	N/A	Respondents reported subjective improvement in pain (67%), appetite (60%), xerostomia (53%), sticky saliva (47%), difficulty chewing (33%), dysphagia (60%), muscle spasm (47%), weight gain/stability (73%), depression (67%) and anxiety (33%). Delivery methods used included smoking (80%), ingestion (27%) and vaporized (20%) with 80% reporting ≥daily use.
Cote et al. 2015 [23]	HNSCC patients undergoing radiotherapy	56	Nabilone	First Week: 0.5mg PO nightly Second Week: 0.5mg PO BID Third Week to End of Treatment: dose adjusted by radio-oncologist to maximum of 0.5mg PO QID	Nabilone was not associated with a statistically significant improvement in quality of life, pain, weight loss, nausea, sleep or mood. However, no adverse effects were observed including drowsiness, anxiety or xerostomia.

Two included studies analyzed the effects of cannabis on survival and disease recurrence [19,20]. The first was a retrospective cohort study [19] investigating the effects of recreational cannabis use on survival outcomes in a group of 74 patients undergoing treatment for oropharyngeal squamous cell carcinoma (OPSCC). Findings of this study indicate that recreational cannabis users had significantly worse overall survival in comparison with non-users (1-year: 60% vs 82% and 3-years: 30% vs 73%; p=0.005), a non-significant detrimental impact on the disease-free state (1-year: 69% vs 77% and 3-years: 27% vs 70%; p=0.051) and no difference in either locoregional recurrence or distant metastasis.

Conversely, a prospective cohort study compared a population of 94 current cannabis users with p16 positive OPSCC patients undergoing treatment with curative intent to a group of non-marijuana users case-matched in a one-to-one scheme based on age, gender, and clinical staging [20]. The study found no significant difference (p=0.400) between cannabis and non-cannabis users in two-year (90% vs 80%) and five-year (80% vs 72%) survival. Moreover, there was no significant difference in rates of overall recurrence or distant metastasis.

Four of the included studies investigated the effects of cannabis or cannabinoids on various quality of life (QOL) measures in head and neck cancer patients [19,21-23]. The results across these trials were mixed. The retrospective study by Ghanem et al. [19] referenced previously not only found decreased survival in cannabis users but also higher median pain scores and greater mean weight loss (22.2 vs 18.5 lbs.) in OPSCC patients undergoing radiotherapy, although these differences failed to meet statistical significance (p>0.05). The only randomized controlled trial included in this review assessed the impact of synthetic cannabinoid nabilone, in a group of 56 head and neck squamous cell carcinoma (HNSCC) patients undergoing radiotherapy [22]. The double-blind study design randomized patients to either receive nabilone or a placebo and assessed participants using three QOL scales (i.e. Organization for the Research and Treatment of Cancer Quality of Life Questionnaire (EORTC QLQ-C30), EORTC QLQ-C30 with specific module for head and neck cancer (H&N35), 10cm Visual Analogue Scale). Nabilone was not found to improve any of the various QOL studied including pain score (p=0.60), analgesic use (p=0.67), appetite (p=0.33) weight loss (p=0.15), nausea (p=0.71), sleep (p=0.44) or mood (p=0.32).

Conversely, a prospective cohort trial by Zhang et al. assessed the impact of recreational loose-leaf cannabis on a population of 148 patients with HNSCC undergoing treatment with curative intent [21]. Recreational cannabis users were case-matched to non-users in a one-to-one scheme based on age, sex, and tumor subsite. Using the two distinct QOL metrics, Edmonton Symptoms Assessment System (ESAS) and the EuroQOL-5D (EQ5D), findings indicated significant improvement in pain (mean [SD], 1.85 [2.49] vs 2.72 [2.59]; difference, 0.87; 95%CI, 0.04-1.69), anxiety (0.77 [1.31] vs 5.30 [2.06]; difference,4.53; 95% CI, 3.97-5.09), depression (0.72 [1.68] vs 3.19[3.05]; difference, 2.47; 95% CI, 1.67-3.27) and general well-being (4.05 [2.29] vs 2.12 [2.65];difference, 1.93; 95% CI, 1.13-2.74) for the cannabis cohort, compared to the control group. There was however no change in mobility, self-care, and usual activity scores between these two groups [21].

Elliott et al. conducted a cross-sectional survey study that included 15 HNSCC patients being treated with either radiotherapy or chemoradiotherapy and enrolled in the Oregon Medical Marijuana Program [22]. This survey utilized four questionnaires (i.e. EORTC QLQ-C30, EORTC QLQ-C30 H&N35, Quality Of Life Radiation Therapy Instrument and the Head and Neck Module (QOL-RTI/HN), Medical Marijuana for Head and Neck Cancer Questionnaire (QOL-HN/MM)). Self-reported patient data suggested that the addition of medicinal cannabis provided subjective improvement in pain (67 %), appetite (60 %), xerostomia (53 %), sticky saliva (47 %), difficulty chewing (33 %), dysphagia (60 %), muscle spasm (47 %), weight gain/stability (73 %), depression (67 %) and anxiety (33 %). However, this study is descriptive in nature, has a notably small sample size, and lacks a control group which limits its overall utility. 

Only the RCT investigating the effects of nabilone on HNSCC patients undergoing radiotherapy reported on the adverse effects of cannabinoid therapy [23]. Nabilone was found to be well-tolerated without obvious adverse impact or increase in drowsiness, anxiety, or xerostomia in comparison to the control group. Notably, none of the studies investigating recreational or medical cannabis use provided data regarding side effects or adverse events.

Only the cross-section survey by Elliot et al. (which utilized the Oregon Medicinal Marijuana Program) reported on the method of cannabis delivery [22]. Patients reported inhaling (n=12; 80%), ingestion (n=4; 27%) or vaporization (n=3; 20%) of cannabis. Eighty percent of respondents used medical cannabis either daily or more than once daily.

In regard to ongoing research, our search identified only one incomplete study registered with ClinicalTrials.gov. This pilot study is a prospective cohort with an estimated population size of 30 participants. The main goal of this study is to determine the adherence and health-seeking behavior of head & neck cancer patients certified to receive cannabis as supportive treatment during chemoradiation therapy. The study plans to stratify patients into groups defined as standard, frail/elderly, and cannabis experienced as defined by more than weekly recreational cannabis use in the past year. The study is currently in the recruitment phase with an anticipated completion date of July 2022 [24].

Discussion

This systematic review has identified a severe deficit in the literature, with only five articles meeting inclusion criteria. These studies were of varying quality (Table 2). While one was an RCT, the use of a synthetic cannabinoid and a small sample size limit the generalizability of these findings [22]. Two trials were prospective cohort series with overlapping study populations, which further limits the sample size of this review [20,21]. Only a single study investigated the effects of a standardized dose of a medical cannabis product [22] with the remainder broadly focusing on ill-defined recreational [19-21] or medical [23] cannabis use. This amount of variability further hinders the ability to draw any consensus. It is thus difficult to draw any meaningful conclusions on the utility/safety of medicinal cannabis/cannabinoids as an adjunctive treatment in head and neck cancer (HNC) patients, with the available literature.

There is limited evidence to suggest any improvement in QOL metrics with the addition of cannabis during HNC treatment. Consequently, it is not prudent to actively recommend utilization without additional evidence. However, it is important to consider how to counsel patients who are actively (or considering) utilizing cannabis either medicinally or recreationally. While one study suggested an impact on overall survival [19], none of the included series demonstrated any significant impact on disease-free survival, locoregional recurrence rates, or distant metastasis. Adverse event reporting was limited, but most patients seem to tolerate cannabis products. While not noted in included studies, there is a suggestion that cannabis can cause drowsiness, dizziness, or a mental slowing which is something patients should consider [25]. 

Although data concerning the role of medicinal cannabis in patients with HNC is scarce, the evidence assessing the application amongst cancer patients generally is somewhat more robust. For example, a systematic review of RCTs comparing multiple doses of cannabinoid extracts administered via any route vs. placebo for the management of cancer-related pain included five studies with a total population size of 1442. Meta-analysis revealed no significant difference between cannabinoids and placebo for mean pain scores across the included studies (mean difference -0.21 [-0.48 to 0.07, p=0.14]). Cannabinoids were associated with significantly higher odds of developing somnolence [26]. Another review identified five RCTs evaluating the impact of various cannabis products. When considering the utility of cannabis products vs. placebos, this meta-analysis failed to identify an improvement in appetite, pain, sleep, or overall quality of life. This analysis however demonstrated the relative safety of cannabinoids without dizziness, alterations in mental health, or other serious adverse treatment effects [27].

Cannabinoids represent a diverse group of pharmacologic agents that vary greatly in their pharmacodynamics [5,28,29]. Although most cannabis-based products contain a wide variety of chemical compounds (i.e., cannabinoids and terpenes) the most common and widely studied are the cannabinoids delta-9-tetrahydrocannabidiol (THC) and cannabidiol (CBD) [12,30]. Cannabinoids exert their effects mainly by binding and activating the Gαi protein-coupled receptors named CB1 and CB2. CB1 is ubiquitous but is found at the highest concentration in the central nervous system where it mediates psychoactive effects while CB2 is mainly concentrated within parts of the immune system [31,32]. THC is the primary psychoactive cannabinoid, acting as a partial agonist at both CB1 and CB2 receptors. It is thus likely responsible for most of the adverse effects associated with cannabis such as drowsiness, dizziness, and mental slowing [33]. One RCT found an association between oral administration of THC and symptoms of anxiety, dysphoria, positive psychotic symptoms, physical sedation, mental sedation, and increased heart rate relative to CBD and placebo [34]. On the other hand, CBD is a non-psychoactive compound and purported to be responsible for the numerous beneficial effects (i.e., analgesic, antiemetic, anti-inflammatory, etc.) ascribed to medicinal cannabis [30,35].

Cannabinoid products can be classified into three categories based on the source of production (Table 1). These include endogenously produced endocannabinoids [36], naturally occurring phytocannabinoids [28], and synthetic cannabinoids [31]. As identified in our review, research utilizing synthetic cannabinoids is often of higher quality, given some ability to standardize drug dosage and delivery. However, the effectiveness of these synthetic cannabinoids has been marginal at best [22,37,38] and there remains wide variability in synthetic products with varying ratios of THC to CBD. This becomes further challenging with plant-derived cannabis products with an innumerable array of differing medicinal strains, formulations, and delivery methods. Consequently, the majority of research has not controlled for the type of cannabinoid or the cannabis strain/cannabinoid composition used by participants [39,40].

Overall, the research into the role of medical cannabis for head & neck cancer patients remains in an early phase possibly due to the recent move towards legalization [41]. It is difficult to make any definitive recommendations to patients regarding the efficacy and safety of medical cannabis products for head & neck cancer patients based on the current literature. At this stage, small pilot studies and proof-of-concept research would be helpful to advance this area of research and pave the way for randomized controlled trials which may help define the role of medical cannabis. Unfortunately, this preliminary research does not seem to be particularly active as, at the time of this review, only a single ongoing trial was registered at ClinicalTrials.gov [24].

For now, when asked about medical cannabis, it would be reasonable to advise patients to avoid high THC products to minimize potential adverse effects [30,33,34]. Patients should also be advised that THC concentrations in cannabis products have risen over time [33,42]. Generally, current literature suggests THC dosing be limited to 30mg/day or less. It is advisable to begin with a low dose in conjunction with CBD and slowly titrate up to avoid adverse effects [43,44]. Additionally, although two meta-analyses failed to find a connection between cannabis use and increased risk of head & neck cancer [45,46], patients should be advised to avoid smoked products as their role as a carcinogen and potential adverse pulmonary effects have yet to be clearly defined [47]. As detailed in Table 1, patients could also be educated about products with a 1:1 ratio of THC:CBD such as nabiximol and those with oral or topical delivery methods [7,48]. Several studies have demonstrated the tolerability of oromucosal nabiximol sprays [49-51].

## Conclusions

The literature regarding the effect of cannabis/cannabinoids on HNC patients undergoing treatment is extremely limited. With only five articles identified, it is difficult to draw any conclusions regarding its safety, effect on QOL, side effects or patterns of usage in the HNC population. More, high-quality studies are necessary for physicians to provide evidence-based advice to HNC patients who are either using or interested in using cannabis as an adjunctive treatment.

## Appendices

Appendix A. PRISMA 2020 checklist

**Table 4:** PRISMA 2020 Checklist PRISMA=Preferred Reporting Items for Systematic Reviews and Meta-Analyses

Section and Topic	Item #	Checklist item	Location where item is reported
TITLE			
Title	1	Identify the report as a systematic review.	Abstract
ABSTRACT			
Abstract	2	See the PRISMA 2020 for Abstracts checklist.	Abstract
INTRODUCTION			
Rationale	3	Describe the rationale for the review in the context of existing knowledge.	Introduction and Background
Objectives	4	Provide an explicit statement of the objective(s) or question(s) the review addresses.	Last paragraph of Introduction and Background
METHODS			
Eligibility criteria	5	Specify the inclusion and exclusion criteria for the review and how studies were grouped for the syntheses.	Methods
Information sources	6	Specify all databases, registers, websites, organisations, reference lists and other sources searched or consulted to identify studies. Specify the date when each source was last searched or consulted.	Methods
Search strategy	7	Present the full search strategies for all databases, registers and websites, including any filters and limits used.	Appendix A
Selection process	8	Specify the methods used to decide whether a study met the inclusion criteria of the review, including how many reviewers screened each record and each report retrieved, whether they worked independently, and if applicable, details of automation tools used in the process.	Methods, Figure 1
Data collection process	9	Specify the methods used to collect data from reports, including how many reviewers collected data from each report, whether they worked independently, any processes for obtaining or confirming data from study investigators, and if applicable, details of automation tools used in the process.	Second paragraph of Methods
Data items	10a	List and define all outcomes for which data were sought. Specify whether all results that were compatible with each outcome domain in each study were sought (e.g. for all measures, time points, analyses), and if not, the methods used to decide which results to collect.	Second paragraph of Methods
	10b	List and define all other variables for which data were sought (e.g. participant and intervention characteristics, funding sources). Describe any assumptions made about any missing or unclear information.	Second paragraph of Methods
Study risk of bias assessment	11	Specify the methods used to assess risk of bias in the included studies, including details of the tool(s) used, how many reviewers assessed each study and whether they worked independently, and if applicable, details of automation tools used in the process.	Second paragraph of Methods
Effect measures	12	Specify for each outcome the effect measure(s) (e.g. risk ratio, mean difference) used in the synthesis or presentation of results.	N/A: no meta-analysis
Synthesis methods	13a	Describe the processes used to decide which studies were eligible for each synthesis (e.g. tabulating the study intervention characteristics and comparing against the planned groups for each synthesis (item #5)).	N/A: no meta-analysis
	13b	Describe any methods required to prepare the data for presentation or synthesis, such as handling of missing summary statistics, or data conversions.	N/A: no meta-analysis
	13c	Describe any methods used to tabulate or visually display results of individual studies and syntheses.	Table 3
	13d	Describe any methods used to synthesize results and provide a rationale for the choice(s). If meta-analysis was performed, describe the model(s), method(s) to identify the presence and extent of statistical heterogeneity, and software package(s) used.	N/A: no meta-analysis
	13e	Describe any methods used to explore possible causes of heterogeneity among study results (e.g. subgroup analysis, meta-regression).	N/A: no meta-analysis
	13f	Describe any sensitivity analyses conducted to assess robustness of the synthesized results.	N/A: no meta-analysis
Reporting bias assessment	14	Describe any methods used to assess risk of bias due to missing results in a synthesis (arising from reporting biases).	N/A: no meta-analysis
Certainty assessment	15	Describe any methods used to assess certainty (or confidence) in the body of evidence for an outcome.	N/A: no meta-analysis
RESULTS			
Study selection	16a	Describe the results of the search and selection process, from the number of records identified in the search to the number of studies included in the review, ideally using a flow diagram.	Figure 1
	16b	Cite studies that might appear to meet the inclusion criteria, but which were excluded, and explain why they were excluded.	Figure 1
Study characteristics	17	Cite each included study and present its characteristics.	Results, Table 3
Risk of bias in studies	18	Present assessments of risk of bias for each included study.	Table 2
Results of individual studies	19	For all outcomes, present, for each study: (a) summary statistics for each group (where appropriate) and (b) an effect estimate and its precision (e.g. confidence/credible interval), ideally using structured tables or plots.	Results, Table 3
Results of syntheses	20a	For each synthesis, briefly summarise the characteristics and risk of bias among contributing studies.	Results, Discussion
	20b	Present results of all statistical syntheses conducted. If meta-analysis was done, present for each the summary estimate and its precision (e.g. confidence/credible interval) and measures of statistical heterogeneity. If comparing groups, describe the direction of the effect.	N/A: no meta-analysis
	20c	Present results of all investigations of possible causes of heterogeneity among study results.	N/A: no meta-analysis
	20d	Present results of all sensitivity analyses conducted to assess the robustness of the synthesized results.	N/A: no meta-analysis
Reporting biases	21	Present assessments of risk of bias due to missing results (arising from reporting biases) for each synthesis assessed.	N/A: no meta-analysis
Certainty of evidence	22	Present assessments of certainty (or confidence) in the body of evidence for each outcome assessed.	N/A: no meta-analysis
DISCUSSION			
Discussion	23a	Provide a general interpretation of the results in the context of other evidence.	Last two paragraphs of discussion
	23b	Discuss any limitations of the evidence included in the review.	First two Paragraphs of Discussion
	23c	Discuss any limitations of the review processes used.	Discussion
	23d	Discuss implications of the results for practice, policy, and future research.	Last two paragraphs of discussion
OTHER INFORMATION			
Registration and protocol	24a	Provide registration information for the review, including register name and registration number, or state that the review was not registered.	First paragraph of methods
	24b	Indicate where the review protocol can be accessed, or state that a protocol was not prepared.	First paragraph of methods
	24c	Describe and explain any amendments to information provided at registration or in the protocol.	N/A
Support	25	Describe sources of financial or non-financial support for the review, and the role of the funders or sponsors in the review.	Disclosures and Acknowledgements
Competing interests	26	Declare any competing interests of review authors.	Disclosures and Acknowledgements
Availability of data, code and other materials	27	Report which of the following are publicly available and where they can be found: template data collection forms; data extracted from included studies; data used for all analyses; analytic code; any other materials used in the review.	N/A

Appendix B. Study protocol

Review Question

Are cannabis and cannabinoid products safe and effective at treating the side effects of head and neck cancer and its treatments in the head and neck cancer population?

Databases

PubMed, Embase, Web of Science, OpenGrey, WONDER, ClinicalTrials.gov

Inclusion Criteria

Population: Head and neck cancer patients, undergoing treatment or post-treatment  

Intervention: Cannabis, cannabinoids

Comparison: Standard of care, approved anti-nausea medication, approved pain medication

Outcomes: Effect on cancer symptoms, effect on cancer treatment symptoms, adverse effects of cannabis use

Exclusion Criteria

Population: Non-head and neck cancer patients, not undergoing treatment or post-treatment

Intervention: Non-cannabis or cannabinoid treatments

Comparison: N/A

Outcomes: Risk of developing primary head and neck cancer

Screening and Data Extraction

Papers will initially be screened on the basis of title and abstract by two, independent reviewers. Only those selected by both will move on to the next stage of review. Any disagreements will be settled by thorough discussion. A second, full-text review will be conducted by a single reviewer and articles presenting relevant data will be included for data collection. A single reviewer will collect any data related to the efficacy of cannabis in treating the symptoms of head & neck cancer and its treatments along with data pertaining to the tolerability of cannabis products. Other data collected will include study design, population size and patient demographics (i.e. cancer type, treatment status, treatment type). All data will be collected using a pre-made data template and reviewed by a separate reviewer. Disagreements will be resolved through thorough discussion.

Study Quality Assessment

A modified National Institutes of Health Study Quality Assessment Tool will be used to assess the methodological quality of included studies and rank them on a scale of zero to eleven. A higher rating will indicate a higher quality study.

Data Synthesis

If sufficient data is collected, we will conduct a meta-analysis. If not, results will be presented in a narrative form.

Appendix C. Search strategy

PubMed

(head and neck cancer [MeSH Terms] OR cancer, head and neck OR cervicofacial cancer OR ear nose throat cancer OR head and neck cancer OR head neck cancer OR otorhinolaryngeal cancer OR otorhinolaryngologic cancer OR otorhinolaryngological cancer OR orl cancer OR ent cancer OR mouth cancer [MeSH Terms] OR cancer, mouth OR intraoral cancer OR mouth cancer OR mouth mucosa cancer OR oral cancer OR oral cavity cancer) AND (cannabis [MeSH Terms] OR cannabis sativa extract OR cannabis sativa leaf OR cannabis sativa resin OR cannabis OR cannabis alkaloid OR cannabis constituent OR cannabis extract OR cannabis herba OR cannabis leaf OR hemp extract OR herba cannabis OR marihuana OR marijuana OR charas OR hashish oil OR hashish OR tetrahydrocannabinol [MeSH Terms] OR 1 trans delta 8 tetrahydrocannabinol OR 7, 8, 9, 10 tetrahydrocannabinol OR delta 2 tetrahydrocannabinol OR delta 3 tetrahydrocannabinol OR delta 5 tetrahydrocannabinol OR delta 6a, 10a tetrahydrocannabinol OR delta tetrahydrocannabinol OR delta3 tetrahydrocannabinol OR delta3 thc OR sp 104 OR tetrahydro cannabinol OR tetrahydrocannabinal OR tetrahydrocannabinol OR tetrahydrocannabinol isomer OR tetrahydrocannabiol OR trans tetrahydrocannabinol OR cannabidiol [MeSH Terms] OR  cannabidiol OR epidiolex OR epidyolex OR gwp42003p OR trans cannabidiol)

Embase

('head and neck cancer'/exp OR 'cancer, head and neck' OR 'cervicofacial cancer' OR 'ear nose throat cancer' OR 'head and neck cancer' OR 'head neck cancer' OR 'otorhinolaryngeal cancer' OR 'otorhinolaryngologic cancer' OR 'otorhinolaryngological cancer' OR 'orl cancer' OR 'ent cancer' OR 'mouth cancer'/exp OR 'cancer, mouth' OR 'intraoral cancer' OR 'mouth cancer' OR 'mouth mucosa cancer' OR 'oral cancer' OR 'oral cavity cancer') AND ('cannabis'/exp OR 'cannabis sativa extract' OR 'cannabis sativa leaf' OR 'cannabis sativa resin' OR 'cannabis' OR 'cannabis alkaloid' OR 'cannabis constituent' OR 'cannabis extract' OR 'cannabis herba' OR 'cannabis leaf' OR 'hemp extract' OR 'herba cannabis' OR 'marihuana' OR 'marijuana' OR 'charas' OR 'hashish oil' OR 'hashish' OR 'tetrahydrocannabinol'/exp OR '1 trans delta 8 tetrahydrocannabinol' OR '7, 8, 9, 10 tetrahydrocannabinol' OR 'delta 2 tetrahydrocannabinol' OR 'delta 3 tetrahydrocannabinol' OR 'delta 5 tetrahydrocannabinol' OR 'delta 6a, 10a tetrahydrocannabinol' OR 'delta tetrahydrocannabinol' OR 'delta3 tetrahydrocannabinol' OR 'delta3 thc' OR 'sp 104' OR 'tetrahydro cannabinol' OR 'tetrahydrocannabinal' OR 'tetrahydrocannabinol' OR 'tetrahydrocannabinol isomer' OR 'tetrahydrocannabiol' OR 'trans tetrahydrocannabinol' OR 'cannabidiol'/exp OR '2 [3 methyl 6 (1 methylethenyl) 2 cyclohexen 1 yl] 5 pentyl 1, 3 benzenediol' OR '2 [3 methyl 6 (prop 1 en 2 yl) cyclohex 2 en 1 yl] 5 pentylbenzene 1, 3 diol' OR 'cannabidiol' OR 'epidiolex' OR 'epidyolex' OR 'gwp 42003p' OR 'gwp42003p' OR 'nabidiolex' OR 'trans cannabidiol')

Web of Science

(head and neck cancer OR cancer, head and neck OR cervicofacial cancer OR ear nose throat cancer OR head and neck cancer OR head neck cancer OR otorhinolaryngeal cancer OR otorhinolaryngologic cancer OR otorhinolaryngological cancer OR orl cancer OR ent cancer OR mouth cancer OR cancer, mouth OR intraoral cancer OR mouth cancer OR mouth mucosa cancer OR oral cancer OR oral cavity cancer) AND (cannabis OR cannabis sativa extract OR cannabis sativa leaf OR cannabis sativa resin OR cannabis OR cannabis alkaloid OR cannabis constituent OR cannabis extract OR cannabis herbal OR cannabis leaf OR hemp extract OR herbal cannabis OR marihuana OR marijuana OR charas OR hashish oil OR hashish OR tetrahydrocannabinol OR 1 trans delta 8 tetrahydrocannabinol OR 7, 8, 9, 10 tetrahydrocannabinol OR delta 2 tetrahydrocannabinol OR delta 3 tetrahydrocannabinol OR delta 5 tetrahydrocannabinol OR delta 6a, 10a tetrahydrocannabinol OR delta tetrahydrocannabinol OR delta3 tetrahydrocannabinol OR delta3 thc OR sp 104 OR tetrahydro cannabinol OR tetrahydrocannabinal OR tetrahydrocannabinol OR tetrahydrocannabinol isomer OR tetrahydrocannabiol OR trans tetrahydrocannabinol OR cannabidiol OR  cannabidiol OR epidiolex OR epidyolex OR gwp42003p OR trans cannabidiol)
